# Oxidative Stress in Vascular Pathophysiology: Still Much to Learn

**DOI:** 10.3390/antiox10050673

**Published:** 2021-04-26

**Authors:** Guillermo Zalba

**Affiliations:** 1 Department of Biochemistry and Genetics, University of Navarra, 31008 Pamplona, Spain; gzalba@unav.es; Tel.: +34-948425600; 2 Navarra Institute for Health Research (IdiSNA), 31008 Pamplona, Spain

Low concentration of reactive oxygen species (ROS) is essential for physiological cellular processes. In contrast, oxidative stress resulting from an imbalance due to overproduction of ROS and/or the deterioration of endogenous antioxidant defenses is implicated in vascular disease, including hypertension and atherosclerosis, the main risk factors for stroke, myocardial infarction (MI), and heart failure. Oxidative stress alters genic expression, causes endothelial dysfunction, promotes remodeling of the extracellular matrix, and exacerbates inflammatory and senescent vascular processes. The main vascular oxidant-generating enzymes include NADPH oxidases, xanthine oxidases, lipoxygenases, mitochondrial oxidases, and nitric oxide synthases (NOS). We still do not fully understand the underlying mechanisms of oxidative stress and the pathological effects that an increased ROS production has in cardiovascular tissue. Unravelling these underlying causes is essential to improve disease therapy. This Special Issue entitled “Oxidative Stress in Vascular Pathophysiology” displays a broad synopsis of the main mechanisms of oxidative stress, its impact on vascular inflammation and dysfunction, the identification of principal ROS-generating enzymes, and the potential benefit of targeting these specific sources of oxidative stress to improve vascular function. In the present issue, we have edited 15 papers encompassing eight reviews and seven original research articles. 

Dubois-Deruyet et al. [1] update the data about the physiological role of low ROS production in the cardiovascular system (cell proliferation, migration, and death), and describe the implications of oxidative stress for cardiovascular diseases. This review focuses its attention on ROS produced by NAPDH oxidase or associated with endothelial or mitochondrial dysfunction, and summarizes new therapeutic strategies potentially involved in cardiovascular protection.

SARS-CoV-2 infection alters mitochondrial dynamics, thus promoting oxidative stress, a pro-inflammatory state, cytokine production, and cell death. Vitamin D decreases ROS generation and normalizes a pro-inflammatory state and cytokine production, improving the prognosis of SARS-CoV-2 infection. In an interesting review, de las Heras et al. [2] deepen the knowledge about the role of mitochondria and vitamin D directly involved in the regulation of oxidative stress and an inflammatory state in SARS-CoV-2 infection.

In a smart review, Tóth et al. [3] summarize the information about the role of ROS in the osteochondrogenic phenotype switch of vascular smooth muscle cells (from contractile to osteoblast/chondrocyte-like cells) and subsequent vascular calcification in different experimental models, and describe the potential of ROS-lowering strategies in the prevention of this deleterious condition.

Vascular oxidative stress triggers cellular and molecular mechanisms, which affect atherothrombotic pathophysiology by promoting the activation of coagulation and endothelial dysfunction. Recent evidence suggests that unbalanced autophagy is related with increased ROS generation and atherosclerosis. In this review, Carresi et al. [4] discuss the role that oxidative stress-induced autophagy plays in endothelial dysfunction associated with the development of atherothrombosis.

In another review, Egea et al. [5] describe the impact of ROS and oxidative stress on the onset and progression of genetic diseases that directly affect connective tissue, focused on the two basic structural molecular components of connective tissue, the ground substance and fibers (collagen and elastic fibers).

On the other hand, a review by Yan et al. [6] discuss the effects of chronic hypoxia on the main enzymatic sources of ROS within the pulmonary vasculature. Authors summarize the ROS-induced functional alterations of various Ca^2+^ and K^+^ channels (regulation of Ca^2+^ influx), and of Rho kinase (responsible for myofilament Ca^2+^ sensitivity), and its role in pulmonary hypertension.

Similarly, hypoxia during gestation is associated with increased incidence of maternal complications of preeclampsia, and may influence the fetal development and subsequent risk of cardiovascular and metabolic disease. Hu and Lubo [7] review the current understanding of hypoxia-induced mitochondrial ROS and their role in placental dysfunction and the pathogenesis of pregnancy complications, and discuss therapies that can target mitochondrial ROS in the placental cells.

In an elegant review of this issue, Martínez-Martinez et al. [8] describe the role played by perivascular adipose tissue, the activation of the renin–angiotensin–aldosterone system and endoplasmic reticulum stress in the vascular dysfunction associated with obesity. This review also highlights the involvement of oxidative stress in this vascular damage.

In an original article, Varona et al. [9] point to phosphodiesterase 4B (PDE4B) as a new therapeutic target for abdominal aortic aneurysm (AAA). The administration of the PDE4 selective inhibitor rolipram to angiotensin II-challenged mice protected against AAA formation. The drug attenuated the rise in vascular oxidative stress induced by angiotensin II, decreased the expression of inflammatory markers as well as the recruitment of inflammatory cells into the vessel wall, and normalized the vascular expression/activity of matrix metalloproteinase 2.

In another article, Rudi et al. [10] show the impact of treatment with the angiotensin converting enzyme (ACE) inhibitor ramipril in MI. In a mouse model of MI, immediate treatment with ramipril reduced cardiac inflammation and the number of circulating inflammatory monocytes, an effect that was accompanied by enhanced retention of hematopoietic stem cells (both granulocyte-macrophage and multipotent progenitors). Long-term ACE inhibition for 28 d limited vascular inflammation and reduced superoxide formation, improving endothelial function and consequently survival of mice.

Metabolic syndrome-mediated heart failure with preserved ejection fraction (HFpEF) is commonly accompanied by left atrial cardiomyopathy. Bode et al. [11] perform an interesting study in cardiomyocytes from ZFS-1 obese rats, a good experimental model exhibiting HFpEF and left atrial cardiomyopathy. In vitro, left atrial cardiomyocytes exhibited mitochondrial-fission, oxidative stress and dysfunctional Ca^2+^ handling. The authors report that anti-inflammatory treatment with IL-10 positively affected dysfunctional Ca^2+^ homeostasis of left atrial cardiomyocytes in HFpEF.

Kim et al. [12] investigate the effect of two stimulators of non-integrin 67-kDa laminin receptor, epigallocatechin-3-gallate and a peptide from the pigment epithelium-derived factor, on brain–blood barrier (BBB) integrity and their underlying mechanisms against vasogenic edema formation induced by epilepsy. Both stimulators attenuated serum extravasation and astroglial degeneration in the rat piriform cortex, reverse regulated BBB permeability, and decreased the expression of aquaporin 4 and the NADPH oxidase subunit p47Phox expression in endothelial cells and astrocytes.

Cortes et al. [13] study the potential crosstalk between NOX5-derived oxidative stress and the unfolded protein response (UPR) in endothelial cells and its impact on cardiovascular pathophysiology. In a transcriptomic study, gene ontology analysis revealed that NOX5 overexpression activates the UPR pathway. Activation of the UPR pathway induces greater apoptosis in endothelial cells. Endothelial-specific NOX5 knock-in mice also displayed changes in the expression of the UPR components’ genes. After MI, UPR gene expression associated with echocardiographic parameters, thus supporting a crosstalk between the NOX5 and UPR pathway in endothelial cells, which might play a relevant role in cardiac pathophysiology.

Santana-Garrido et al. [14] evaluate the therapeutic powder of a wild olive oil-enriched diet for the treatment of ocular oxidative stress induced by hypertension. The authors investigate the beneficial effects of ACEBUCHE, the oil obtained from the traditionally cultivated olive tree counterpart. Hypertensive mice obtained by administration of NG-nitro-L-arginine-methyl-ester are subjected to a dietary supplementation with either ACEBUCHE oil or control extra virgin olive oil. The use of ACEBUCHE oil resulted in better outcomes, compared with reference olive oil, against hypertension-related oxidative retinal damage. Interestingly, the ACEBUCHE oil-enriched diet reduced NADPH oxidase activity and expression, mainly the NOX2 isoform, and improved nitric oxide bioavailability and antioxidant enzyme profile in the retinas of hypertensive mice. 

Kefir products possess anti-inflammatory and anti-hypertensive activities and may exert immunomodulatory functions. Chen et al. [15] evaluate the potential beneficial effects of kefir peptides (KPs) on salt-induced renal damage in aged stroke-prone spontaneously hypertensive (SHRSP) rats. Aged SHRSP rats under induction with salt showed multiple renal injuries with increased renal inflammation, fibrosis, oxidative stress, tubular atrophy, and glomerulosclerosis. KPs treatment ameliorates salt-induced renal damage, tubular atrophy, and glomerular dysfunction through anti-inflammatory (reduced infiltration of inflammatory cells), antioxidative stress (diminished ROS production and increased superoxide dismutase activity), and antifibrotic activities diminish levels of transforming growth factor-β). These data highlight the potential use of KPs as a protective agent against high salt-induced renovascular-related diseases.
